# A Sustainable Natural-Rubber IoT Smart Insole for Remote Body-Load Monitoring: An Observational Gait Comparison in Flexible Flatfoot

**DOI:** 10.3390/s26185687

**Published:** 2026-09-08

**Authors:** Prachid Saramolee, Praphatson Sengsoon, Sarawuth Chaimool, Khamphong Khongsomboon, Jakrawat Budboonchu, Siraporn Sakphrom

**Affiliations:** 1 School of Engineering and Technology, Walailak University, Nakhon Si Thammarat 80160, Thailand; sprachid@mail.wu.ac.th; 2 School of Allied Health Sciences, Walailak University, Nakhon Si Thammarat 80160, Thailand; praphatson.kl@wu.ac.th; 3 Department of Electrical Engineering, Faculty of Engineering, Khon Kaen University, Khon Kaen 40002, Thailand; sarachai@kku.ac.th; 4 Department of Electronic and Telecommunication Engineering, National University of Laos, Vientiane P.O. Box 3166, Laos; khongsomboon@fe-nuol.edu.la; 5 Faculty of Engineering, Rajamangala University of Technology Isan Khonkaen Campus, Khon Kaen 40000, Thailand; jakrawatb@gmail.com; 6 Center of Excellence in Sustainable Disaster Management, Walailak University, Nakhon Si Thammarat 80160, Thailand

**Keywords:** smart insole, natural rubber composite, wearable sensors, Internet of Things, remote patient monitoring, gait analysis, flexible flatfoot, plantar load

## Abstract

Flexible flatfoot (pes planus) alters lower-limb biomechanics and plantar-pressure distribution, raising the risk of pain and injury. Laboratory gait analysis with optical motion capture and force plates is the reference standard but is costly, space-constrained, and ecologically limited. We present the design, fabrication, and validation of a low-cost, sustainable smart insole for Internet-of-Things (IoT) remote body-load monitoring. The device pairs a dual-layer natural-rubber body—a silica-filled sponge–rubber upper for comfort and a carbon-black-reinforced solid outsole for durability—with four load cells per insole at high-pressure plantar landmarks, read through a 24-bit amplifier by an ESP32 that calibrates and streams left/right load over Wi-Fi to the ThingSpeak cloud, with a wrist-worn OLED for real-time feedback. Against reference weights in 25 participants, the system measured total body weight with a mean absolute error of 2.94%, a maximum error of 4.18%, and an RMSE of 1.94 kg (Pearson r = 0.99); the residual was an almost purely systematic proportional bias (slope 0.966, R^2^ = 0.98) removable by a single in-sample scalar recalibration. In 30 adults (15 normal-arch; 15 flexible flatfoot), spatiotemporal gait parameters were compared while both groups wore the smart insole. Forward-progression parameters, including step length, stride length, and walking velocity, did not differ significantly between groups during comfortable walking (all *p* > 0.18). The flatfoot group showed a wider mediolateral base—greater stance width during standing (+14%, *p* = 0.008) and step width during comfortable walking (+23%, *p* = 0.040, uncorrected). After correction for multiple comparisons, only the reduction in fast-walking cadence remained statistically significant. A sustainably sourced, affordable smart insole can thus deliver clinically meaningful remote body-load monitoring. The findings also point to a dissociation: forward propulsion was comparable between the groups while the insole was worn, whereas the mediolateral base remained wider in flatfoot. Controlled trials pairing orthotic support with active gait retraining are therefore warranted.

## 1. Introduction

Pes planus (flatfoot) is a prevalent deformity defined by partial or total collapse of the medial longitudinal arch under load [1]. The arch is an elastic structure of ligaments, tendons, and fascia that dissipates weight-bearing forces and stores and releases mechanical energy across the gait cycle [1,2]. In flexible flatfoot, the arch is present when unloaded but collapses on standing, triggering compensations throughout the kinetic chain—excessive rearfoot eversion, increased talar plantarflexion, and forefoot abduction [2,3,4,5]—that reduce shock absorption and raise the risk of pain and injury at the foot, knee, hip, and lumbar spine [1,4]. Symptomatic cases are commonly managed with orthoses and footwear that support the arch and restore alignment [1,3].

Quantitative gait assessment underpins diagnosis, treatment evaluation, and biomechanical understanding. The reference standard—three-dimensional optical motion capture (OMC) with force platforms [6,7]—is expensive, requires dedicated laboratories and trained staff, and confines participants to a small capture volume that can distort natural gait [6,8]. Wearable technology overcomes these constraints: inertial measurement units (IMUs) and pressure-sensitive smart insoles capture gait data unobtrusively and at low cost in real-world settings, enabling continuous, ecologically valid monitoring [9,10,11,12]. Coupling such sensors with the Internet of Things (IoT) links devices to cloud platforms for real-time collection, transmission, and analysis of health data, supporting data-driven care without frequent in-person visits [13,14]; services such as ThingSpeak provide the infrastructure to aggregate, visualize, and analyze these live streams [15,16].

Despite this progress, integrated low-cost systems validated for a specific clinical population and built from sustainable, economically relevant materials remain scarce. This study addresses that gap through three contributions. First, material innovation: The insole is fabricated from natural-rubber composites, exploiting rubber’s favorable footwear mechanics while adding value to a primary Thai commodity, in line with sustainable footwear biocomposites [17] and supported by the Rubber Authority of Thailand. Second, system integration: A complete end-to-end pipeline, from the custom insole and low-cost embedded electronics to cloud visualization and storage. Third, clinical application: The system is used in individuals with flexible flatfoot, yielding observational evidence on their gait while a supportive insole is worn and on the apparent interplay between mechanical support and neuromuscular adaptation.

This study had four objectives: (i) to design and fabricate a comfortable, functional insole from natural-rubber composites capable of integrating a sensor system; (ii) to develop a simple, low-cost sensor system for measuring per-foot body load; (iii) to validate the system’s accuracy against known body weight and biomechanical principles of foot function during standing and walking; and (iv) to compare spatiotemporal gait parameters between individuals with normal feet and flexible flatfoot during standing, comfortable walking, and fast walking while wearing the smart insole.

## 2. Materials and Methods

**Figure 1:**
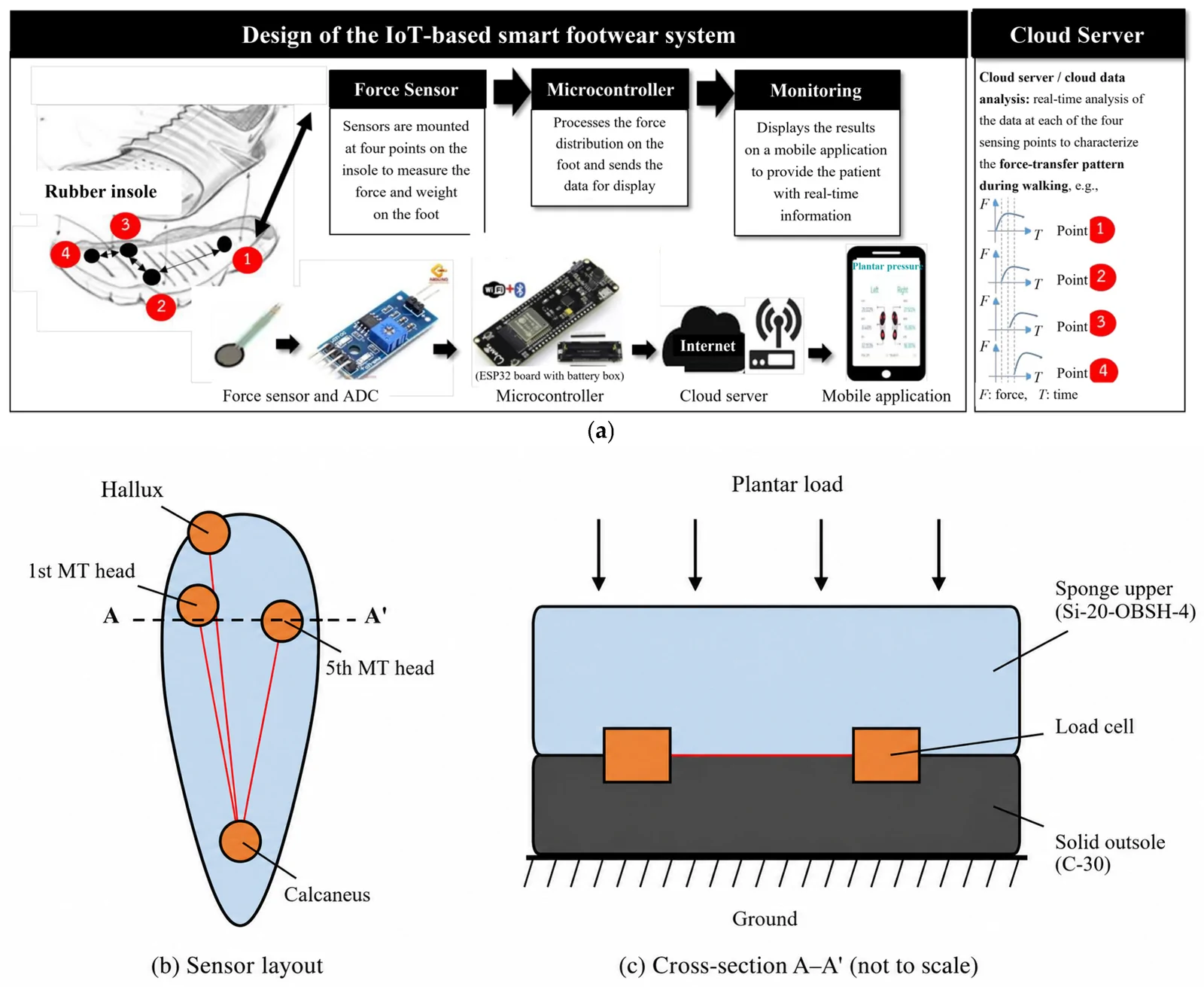
System concept and sensor–insole construction. (**a**) Conceptual design of the IoT-based smart footwear system: Plantar load is sensed at four points on the natural rubber insole, conditioned by a force-sensing and analog-to-digital conversion stage, and processed by an ESP32 microcontroller that streams the data over Wi-Fi to a cloud server for real-time monitoring on a wrist-worn display and web dashboard. (**b**) Plan view of the four load-cell locations on the insole. In the cloud-server panel, each inset plots the force–time response (F, force; T, time) at one of the four sensing points; solid curves show the measured force profile and dashed vertical lines mark [AUTHORS: please specify what the dashed lines denote, e.g., gait-event boundaries]. (**c**) Cross-section A–A′ of the dual-layer stack, showing the load cells embedded between the silica-filled sponge upper (Si-20-OBSH-4) and the carbon-black-reinforced solid outsole (C-30).

This section details the materials, fabrication, electronic design, and clinical validation protocol. Figure 1a shows the complete sensing-to-cloud pipeline.

### 2.1. Smart Insole Fabrication

#### 2.1.1. Design and Prototyping

**Figure 2:**
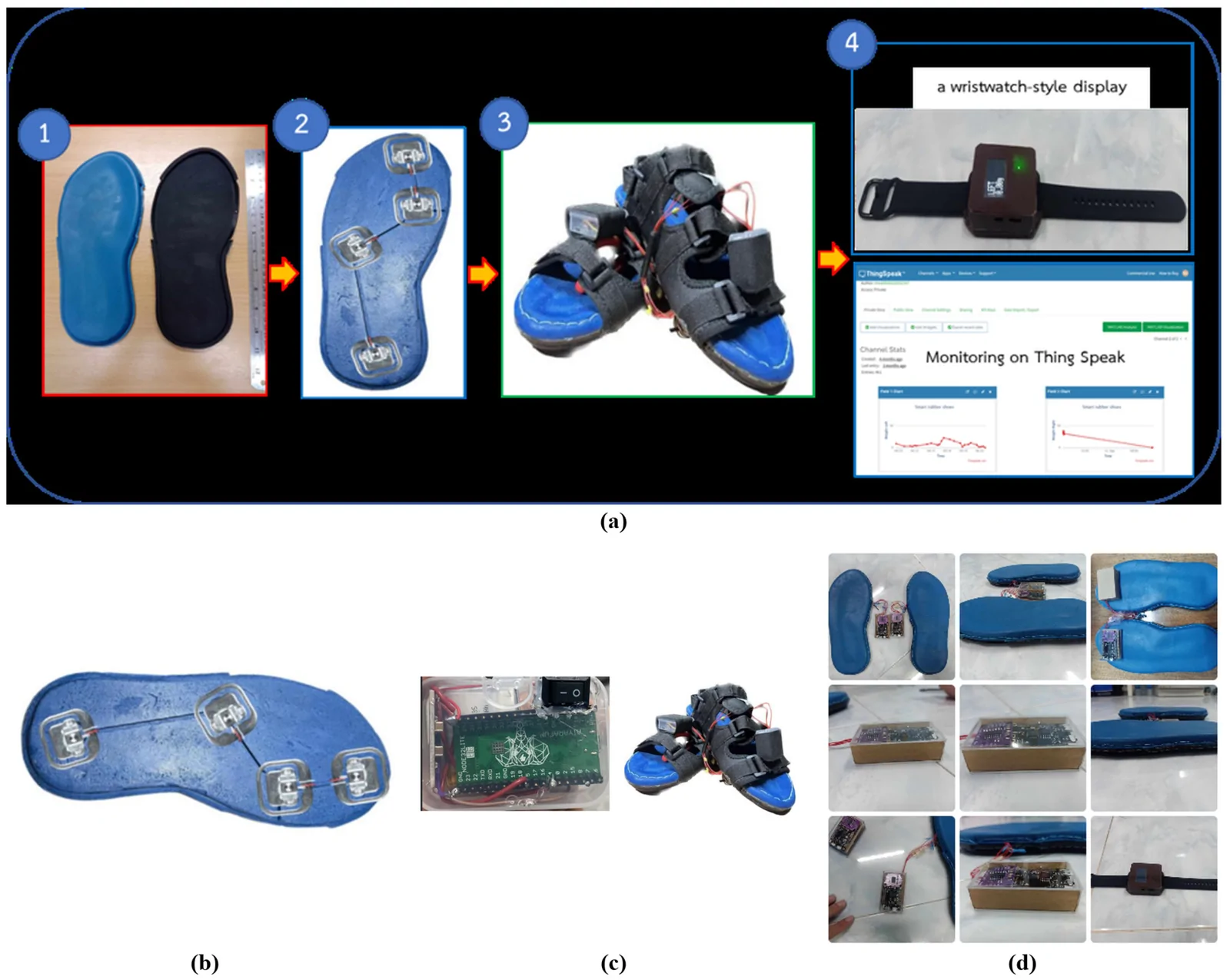
Fabrication and integration of the smart insole. (**a**) Development workflow: Compression-molded natural rubber soles, sensor embedding, footwear assembly, and the wrist-worn display with the cloud dashboard; (**b**) instrumented insole with four load cells at the plantar sites of highest pressure; (**c**) ESP32 and sensor interface in the 4.5 × 4.5 × 2 cm enclosure together with the complete instrumented footwear; and (**d**) stages of hardware integration.

Design began with an analysis of foot morphology: the dry-footprint method captured imprints from individuals representing three arch types—normal, pes planus (flatfoot), and pes cavus (high arch)—informing a universal design with a subtle medial longitudinal arch support adaptable across foot types. Prototypes were 3D-printed to refine size, shape, and ergonomics, after which industrial-grade steel molds were manufactured for compression molding. Figure 2a summarizes the resulting workflow—from compression-molded rubber soles, through sensor embedding and footwear assembly, to the wrist-worn display and cloud dashboard—detailed in the following subsections.

#### 2.1.2. Rubber Composite Formulation

The insole used two natural-rubber composites, each formulated for a specific function (Table 1). To ensure comfort and reduce mass—critical after early prototypes proved to be too heavy—the foot-contacting upper was a sponge–rubber composite (Si-20-OBSH-4):natural rubber (Standard Thai Rubber, STR 5L) with 20 phr silica (Tokusil 255G; Tokuyama Corp., Tokyo, Japan) and 4 phr of the blowing agent oxy-bis-(benzenesulfonyl hydrazide) (OBSH) for a porous, lightweight structure. The outsole, designed for durability, abrasion resistance, and anti-slip performance (C-30), used STR 5L with 30 phr HAF N-330 carbon black, a standard reinforcing filler.

#### 2.1.3. Curing and Mechanical Testing

Compounds were mixed on a two-roll mill and vulcanized by compression molding in a hot press at 150 °C, with cure time set from the t_90_ value (time to 90% of maximum torque) by oscillating disc rheometry (ODR) [18]. Mechanical properties were tested to standards: hardness (Shore A) [19]; tensile strength and elongation at break [20]; tear strength [21]; and heat-aging resistance (70 ± 1 °C for 72 h before re-testing) [22].

### 2.2. Embedded and IoT System Design

#### 2.2.1. Design Requirements and System Overview

The electronic subsystem was engineered against four requirements: minimal unit cost, low mass and unobtrusive form factor, accuracy sufficient for body-load monitoring, and native cloud connectivity. These drove a three-tier wireless sensor-node architecture (Figure 1a): a sensing tier at the insole; an ESP32-based acquisition-and-communication tier; and a cloud tier for storage, visualization, and export, complemented by a wrist-worn feedback unit. Principal components are summarized in Table 2.

#### 2.2.2. Force-Sensing Subsystem

The sensing tier was refined over two generations. Early prototypes usedFlexiForce piezoresistive sensors (Tekscan, Inc., Norwood, MA, USA), whose force-dependent resistance was converted to a voltage by a resistance-to-voltage module and read by the ESP32’s 12-bit ADC. Because load cells offer superior repeatability, lower drift, and a near-linear response under sustained static load—essential for reliable body-weight estimation—the final device used four strain-gauge load cells per insole, embedded between the sponge upper and solid outsole (mechanically protected yet load-coupled) at the four highest-pressure plantar regions: the calcaneus, the medial and lateral metatarsal heads, and the hallux (Figure 2b). These sites reflect the physiological load distribution in quiet standing, where rear- and forefoot each bear roughly half the load, and the forefoot share is divided mainly between the hallux and lateral toes. Within each insole, the four cells form a single full Wheatstone bridge read by one HX711 24-bit amplifier (Avia Semiconductor, Xiamen, China), which reports that foot’s total load to the ESP32 over a two-wire (data/clock) interface. These positions were chosen anatomically rather than by numerical optimization, so that the transducers remain under load in every posture and the summed bridge output tracks total per-foot load. Because the four cells of a foot are wired as a single Wheatstone bridge, the device provides one load channel per foot; cross-talk between channels therefore cannot arise, but the compliant rubber does spread load laterally, so the fraction of the applied load reaching each transducer depends on where the load is applied. This load-spreading term dominates the residual error budget (σ_mech_ in Equation (5)), and position-resolved loading tests, together with an individually wired configuration, are required to quantify it.

#### 2.2.3. Embedded Processing and Measurement Model

The ESP32 module (ESP-32 Mini32 V2.0.13; Espressif Systems, Shanghai, China) is the acquisition and communication core: a Tensilica LX6 dual-core processor at 240 MHz with 520 KB SRAM, Wi-Fi (802.11 b/g/n) and Bluetooth 4.2 BLE, twelve ADC channels, and I^2^C/SPI/UART interfaces, drawing only microamperes in sleep—an efficient balance for battery-powered wearables. Firmware (Arduino IDE; Arduino S.r.l., Ivrea, Italy) reads the per-foot load from each HX711; applies a two-stage calibration—(i) a tare removing the unloaded offset and (ii) a linear scale factor from regression against reference weights; and reports left and right values. A consistent measured-vs-true bias observed during validation was corrected by tuning the calibration factor, giving the sub-5% accuracy of Section 3.2. The microcontroller, amplifier, and display were mounted on a compact board in a 4.5 × 4.5 × 2 cm enclosure (Figure 2c); the integration stages—wiring the electronics to the insole, fitting the board into the enclosure, and layering the instrumented insole between sponge upper and solid outsole—are shown in Figure 2d.

The sensing chain maps applied plantar load to a digital body-load estimate through four cascaded stages that constitute the device measurement model. In each insole, the four strain-gauge elements form a single full Wheatstone bridge whose differential output is proportional to the net gauge strain, as shown in Equation (1):

(1) $$V_{o}=\left(V_{ex}\cdot GF/4\right)\left(\varepsilon_{1}-\varepsilon_{2}+\varepsilon_{3}-\varepsilon_{4}\right)$$

where $V_{ex}$ is the bridge excitation, GF is the gauge factor, and $\varepsilon_{i}$ is the strain at gauge i. For the assembled transducer, this reduces to a linear map, $V_{o,f}=S\cdot F_{f}$, between the bridge output of foot (f) and its total plantar load ($F_{f}$), with S as the lumped sensitivity. The HX711 amplifier digitizes this output at 24-bit resolution with a programmable gain G = 128, as shown in Equation (2):

(2) $$N_{f}=round\left(2^{24}\cdot G\cdot V_{o,f}/V_{ref}\right)$$

Firmware converts the raw count to force through a two-point calibration—a tare term, $N_{0’f}$, that removes the unloaded offset and a scale factor, α, obtained by linear regression against reference masses (Equation (3))—and the measured body load is the sum of the two per-foot channels (Equation (4)):

(3) $$F_{f}=\alpha \left(N_{f}-N_{0,f}\right)$$

(4) $$BW_{meas}=F_{L}+F_{R}$$

Equation (4) defines total body load as the sum of the two per-foot channels, which the firmware reproduced in the acquired data ($BW_{meas}\equiv F_{L}+F_{R}$). Two design consequences follow. First, conversion resolution far exceeds the required accuracy: the least-significant bit is ~0.01 g of the ≈150 kg full scale, and even at the HX711’s ≈ 20 noise-free bits, the effective resolution stays below 0.3 g—orders of magnitude below the observed error. The measurement error therefore decomposes as Equation (5), with the quantization term, $\sigma_{q}$, negligible and the residual dominated by mechanical coupling through the compliant rubber ($\sigma_{mech}$) and calibration linearity ($\sigma_{cal}$):

(5) $${\sigma_{BW}}^{2}={\sigma_{q}}^{2}+{\sigma_{mech}}^{2}+{\sigma_{cal}}^{2}$$

Second, the device showed a small, highly consistent proportional underestimation (measured-to-true gain k ≈ 0.97; per-participant error SD only 0.65%), so a single scalar recalibration (Equation (6)) restores agreement and reduces the mean absolute error from 2.94% to 0.47% (an in-sample estimate; Section 3.2)

(6) $$BW_{corr}=BW_{meas}/k,\;k\approx 0.97$$

#### 2.2.4. Wireless Communication and Cloud Architecture

**Figure 3:**
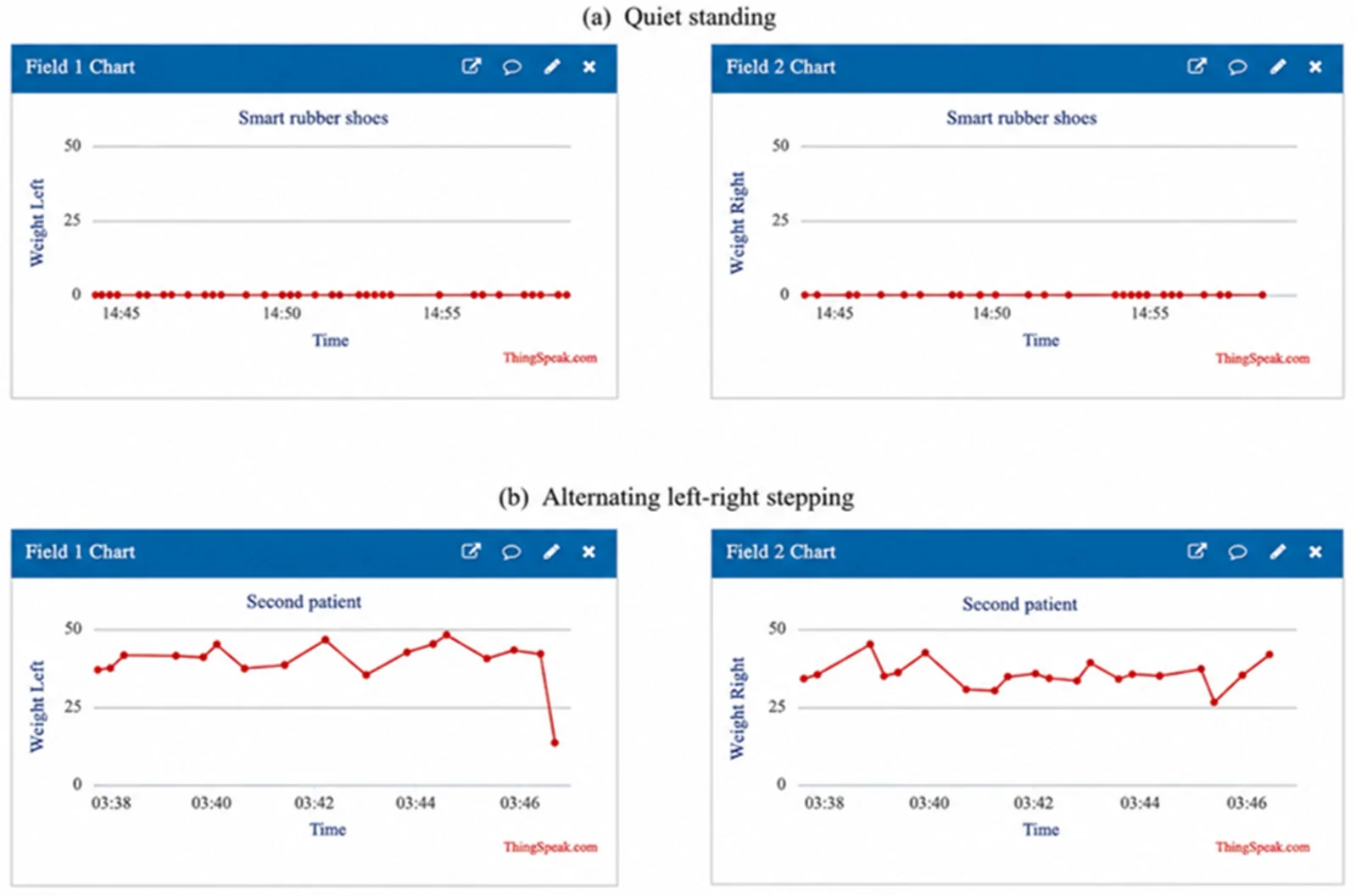
ThingSpeak cloud dashboard showing real-time per-foot load, with Field 1 (left foot) and Field 2 (right foot) plotted against time. (**a**) Quiet standing, with negligible load registered; and (**b**) alternating left–right stepping during walking, showing the per-foot weight-bearing time series.

After on-board processing, the ESP32 joins the local Wi-Fi network and transmits the calibrated left- and right-foot loads to the ThingSpeak IoT platform [15,16]. A dedicated channel aggregates the stream in two fields—Field 1 for the left insole and Field 2 for the right—renders real-time plots, retains the time series, and exports the records (e.g., as CSV/Excel) for offline analysis. The HX711 digitizes samples locally at 10–80 samples/s, but cloud uploads occurred every 15 s, the minimum interval of the platform’s free tier, which is sufficient for tracking weight-bearing trends and adherence to prescribed loading but not for intra-stride event detection, a limitation discussed in Section 4.3. Because the pipeline is transport-agnostic, it can be migrated to a lightweight publish/subscribe protocol (MQTT) or a private broker to support higher sampling rates or clinical-grade deployments. Representative dashboard output for quiet standing and for alternating left–right stepping is shown in Figure 3.

#### 2.2.5. Feedback and Power Management

A 0.96-inch OLED over I^2^C, in a wristwatch-style housing, shows per-foot load in real time, letting a user or clinician check weight distribution during standing and walking without the cloud dashboard. The node is powered by a rechargeable 3.7 V, 370 mAh lithium-polymer cell (502530), and duty-cycling of acquisition and radio activity keeps average current low enough to complete a full assessment session on one charge.

#### 2.2.6. Accuracy Validation Protocol

For accuracy, 25 adults of known body weight stood on the insoles while their calibrated total load ($BW_{meas}$, Equation (4)) was compared with a certified-scale reading ($BW_{true}$). Per participant, we computed the absolute percentage error (Equation (7)); cohort accuracy was summarized by the mean and maximum absolute percentage error (Equation (8)), the RMSE in kg (Equation (9)), the Pearson correlation (Equation (10)), and an ordinary-least-squares calibration line of measured vs. true weight (Equation (11)). The capacity to resolve left–right load asymmetry was quantified by a weight-bearing symmetry index (Equation (12)).

(7) $$e_{i}=\left|BW_{true,i}-BW_{meas,i}\right|/BW_{true,i}\times 100$$

(8) MAPE=(1/n)Σe_i=2.94%, e_max=4.18%

(9) $$RMSE=\sqrt{\left[\left(1/n\right)\Sigma {\left(BW_{meas,i}-BW_{true,i}\right)}^{2}\right]}=1.94\;kg$$

(10) $$r=cov\left(BW_{meas},BW_{true}\right)/\left(\sigma_{meas}\cdot \sigma_{true}\right)=0.990$$

(11) $$BW_{meas}=0.966\cdot BW_{true}+0.28\;\left(R^{2}=0.980\right)$$

(12) $$SI=\left(F_{L}-F_{R}\right)/\left[½\left(FL+FR\right)\right]\times 100$$

### 2.3. Clinical Validation Protocol

#### 2.3.1. Participants

Thirty volunteers participated. After screening, they were divided into two equal groups (*n* = 15): a control group with normal foot posture and an experimental group with flexible flatfoot (pes planus). This study was conducted in accordance with the Declaration of Helsinki; all participants provided written informed consent, and the protocol was approved by the institutional human research ethics committee of Walailak University (approval no. WUEC-22-188-02).

#### 2.3.2. Experimental Procedure

**Figure 4:**
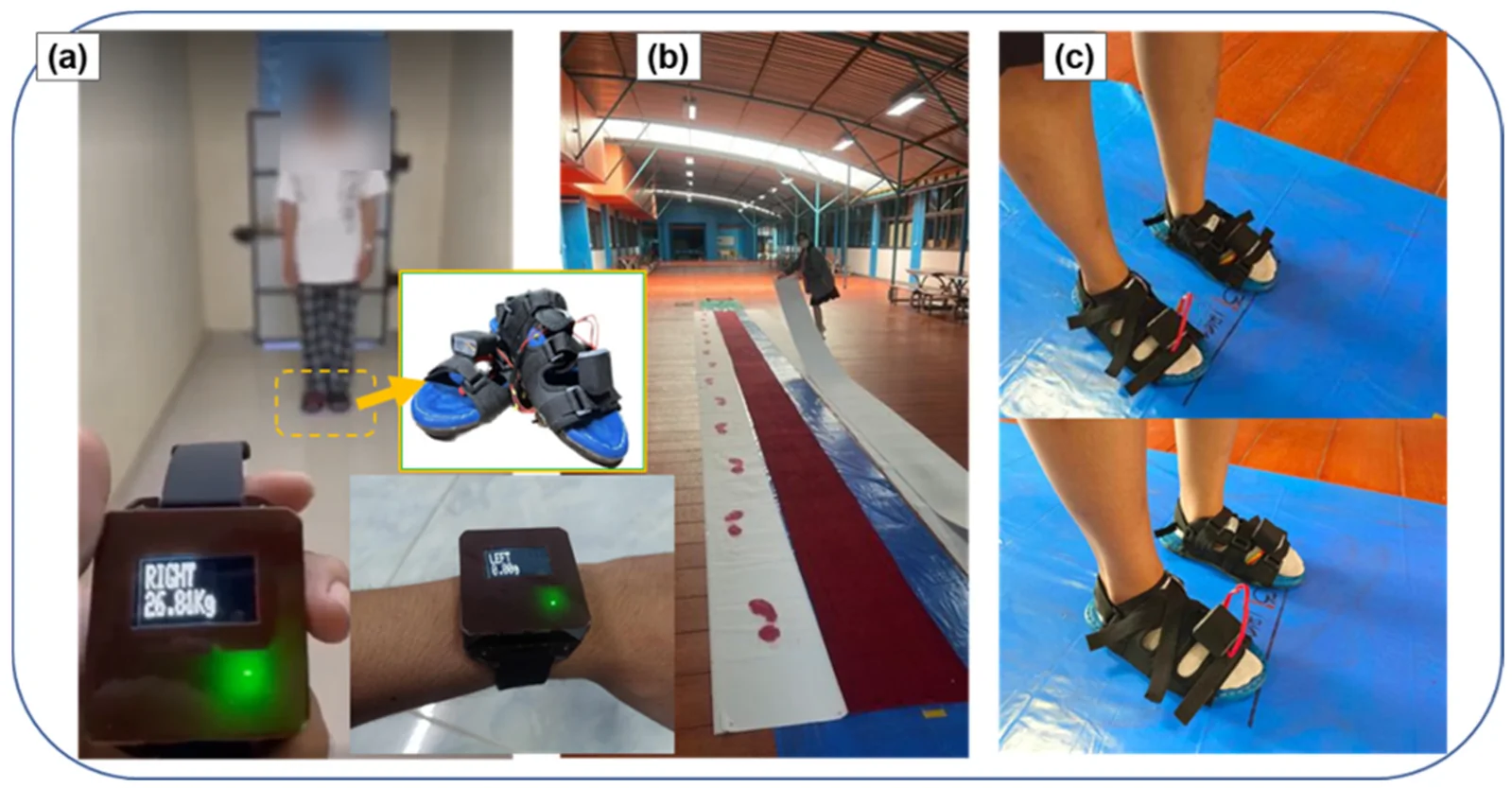
Clinical validation setup. (**a**) A participant during quiet standing while the wrist-worn display reports the calibrated load of each foot in real time; (**b**) the 10 m paper-covered walkway with ink footfall imprints used to extract spatiotemporal gait parameters; and (**c**) a participant wearing the instrumented natural rubber footwear on the test surface.

Spatiotemporal gait parameters were obtained using the dry-footprint method, in which participants stepped onto an ink-saturated pad and walked across a paper-covered 10 m walkway, leaving clear footfall imprints. Although this method does not provide the kinetic information of force plates or the three-dimensional kinematics of optical motion capture, it is a well-established, low-cost technique for measuring fundamental spatiotemporal gait parameters. Throughout all assessments, participants wore the developed smart rubber insoles to monitor per-foot body load. Testing was performed under three conditions: quiet standing, self-selected comfortable walking, and self-selected fast walking. Each condition was preceded by a familiarization trial and repeated three times. Figure 4 shows the setup: per-foot loads verified on the wrist-worn display during standing (a), footfall imprints along the walkway during walking (b), and a participant wearing the instrumented footwear (c). During all trials, the acquisition enclosure (4.5 × 4.5 × 2 cm) was strapped to the dorsum of the footwear rather than beneath the foot, and the display was worn on the wrist (Figure 2c,d and Figure 4c), so the module did not contact the plantar-sensing region. Because both groups wore identical instrumentation, this added mass—noted as a gait confounder in Section 4.3—did not bias the between-group comparison, although it may have influenced the absolute gait values.

#### 2.3.3. Measured Gait Parameters

The following spatiotemporal gait parameters were obtained from the footprint recordings: step length (cm), defined as the distance between the heel strike of one foot and the subsequent heel strike of the contralateral foot; stride length (cm), defined as the distance between two consecutive heel strikes of the same foot; step width (cm), defined as the mediolateral distance between the heel centers of consecutive footprints; cadence (steps/min); and walking velocity (m/s), computed independently as the distance walked divided by the elapsed time. Because these quantities are kinematically linked, velocity is related to step length and cadence through Equation (13) (step length in m; cadence in steps·min^−1^) and is included only to illustrate this relationship.

(13) $$v=L_{step}\cdot C/60$$

#### 2.3.4. Statistical Analysis

For each participant and parameter, the mean and standard deviation (SD) were computed across the three repetitions. To assess measurement quality, inter-rater and intra-rater reliability were evaluated among researchers, yielding high intraclass correlation coefficients (ICC, 0.88–0.94) and thereby indicating consistent, reproducible measurements. Group-level comparisons between the normal-foot and flexible-flatfoot groups were then made for each parameter across the three conditions, and results are reported as mean ± SD. Between-group differences were additionally quantified by the standardized effect size (Cohen’s d, Equation (14)) and tested with Welch’s unequal-variance t statistic (Equation (15)); the resulting values for every parameter and condition are reported in Section 3.4. Because 11 between-group comparisons were performed, significance was additionally interpreted in light of a Holm–Bonferroni correction, and standardized effect sizes—which do not depend on the number of tests—were emphasized; non-significant differences are reported as such and are not interpreted as evidence of equivalence.

(14) $$d=\left(\mu_{1}-\mu_{2}\right)/s_{p}$$

(15) $$t=\left(\mu_{1}-\mu_{2}\right)/\sqrt{\left(s_{1}^{2}/n_{1}+s_{2}^{2}/n_{2}\right)}$$

## 3. Results

### 3.1. Material Properties and Device Characteristics

**Figure 5:**
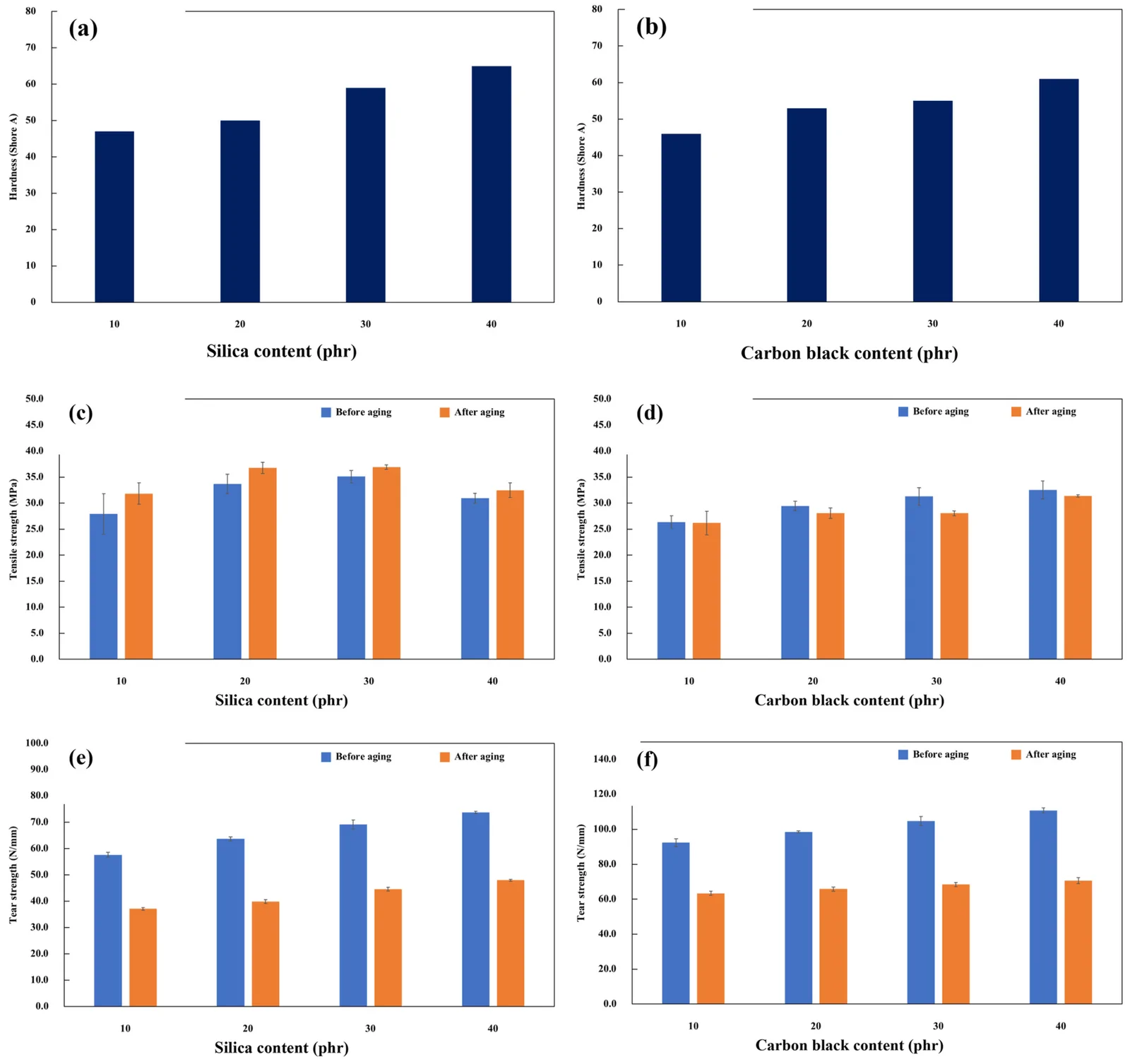
Mechanical properties of the vulcanized natural-rubber compounds as a function of filler loading (10–40 phr): (**a**,**b**) hardness, (**c**,**d**) tensile strength, and (**e**,**f**) tear strength for the silica-filled (**a**,**c**,**e**) and carbon-black-filled (**b**,**d**,**f**) series. Tensile and tear data are shown before and after accelerated heat aging (ISO 188; 70 °C, 72 h). Error bars denote one standard deviation.

The two natural-rubber compounds were characterized for cure behavior and mechanical properties (Table 3). For both fillers, hardness, 300% modulus, tensile strength, and tear strength increased with filler loading, while elongation at break decreased, consistent with the reduced chain mobility expected at higher filler content. On this basis, 20 phr silica (Si-20) was selected for the foot-contacting upper and 30 phr carbon black (C-30) for the outsole. At these loadings the carbon-black outsole was harder and far more tear-resistant, suiting it to abrasion and wear, whereas the silica compound gave a softer, higher-elongation upper more appropriate for comfort. Heat aging (ISO 188; 70 °C, 72 h) altered the properties only modestly, indicating adequate durability. Figure 5 shows the full filler-loading series (10–40 phr) for hardness, tensile strength, and tear strength of both compounds before and after accelerated heat aging. Stress–strain curves and compression properties were outside the characterization set used to select the formulations and are therefore not reported here.

Because early solid-rubber prototypes were too heavy (~520 g per shoe), the upper was converted to a sponge structure by adding 4 phr of the OBSH blowing agent (Si-20-OBSH-4), which lowered its density to ≈0.39 g/cm^3^; blowing-agent contents above 4 phr produced no further density reduction and were therefore not used. The resulting dual-layer construction yielded a comfortable yet durable device of ~400 g per shoe.

### 3.2. System Accuracy and IoT Performance

**Figure 6:**
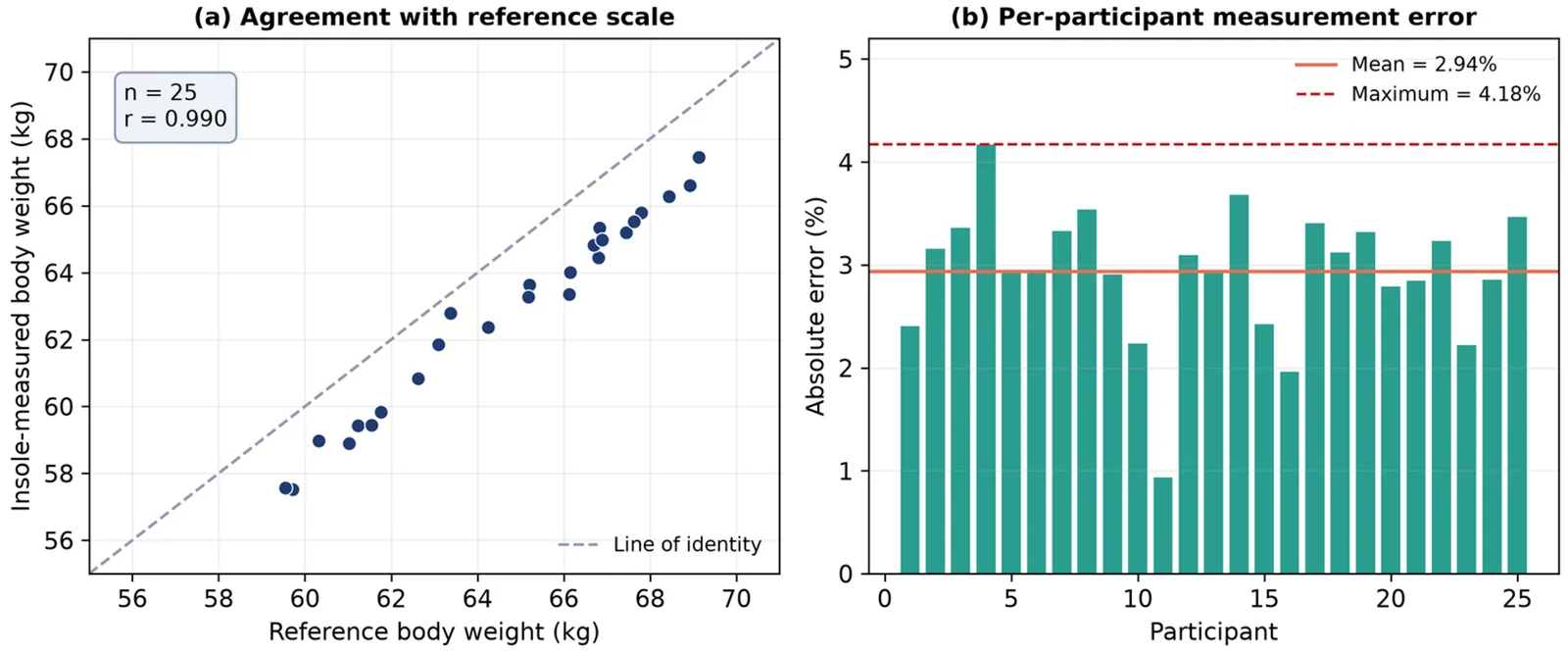
System accuracy for total body-weight measurement (*n* = 25). (**a**) Insole-measured versus reference scale weight, with the dashed line of identity (Pearson r = 0.99); points fall slightly below identity, indicating a small, consistent underestimation. (**b**) Absolute percentage error per participant, with mean (2.94%) and maximum (4.18%) indicated.

The integrated system met its accuracy target with an essentially systematic, correctable error. Across the 25-participant cohort, insole-measured total body weight was strongly correlated with the reference scale (Pearson r = 0.990; Figure 6a) and followed the calibration line $BW_{meas}=0.966\cdot BW_{true}+0.28$ ($R^{2}=0.980$; Equation (11)), giving a mean absolute error of 2.94%, a maximum error of 4.18% (Figure 6b), and a root-mean-square error of 1.94 kg. The near-unity slope and small intercept indicate a proportional underestimation of about 3% (gain k ≈ 0.97) rather than random scatter; because the per-participant error had a standard deviation of only 0.65%, the single scalar correction of Equation (6) reduces the mean absolute error to 0.47% (an in-sample estimate). Consistent with the error budget of Equation (5), this residual is set by mechanical load transfer through the compliant rubber and by calibration linearity—not by the converter, whose effective resolution (<0.3 g) is more than three orders of magnitude finer than the measurement error. The per-foot channels also resolved left–right weight-bearing imbalance, with a mean absolute asymmetry of 8.8% across the cohort (Equation (12)); because each foot uses a separate amplifier and scale factor, part of this apparent asymmetry may reflect inter-channel calibration differences rather than true physiological asymmetry, so per-channel validation against equal reference loads is needed. Calibrated left/right loads streamed to the ThingSpeak dashboard in real time throughout testing, confirming end-to-end operation of the IoT pipeline (Figure 3). These results establish the device as a quasi-static, per-foot body-load monitor rather than a measure of dynamic gait kinetics or regional plantar-pressure distribution. Key indicators are summarized in Table 4.

### 3.3. Participant Demographics

Table 5 summarizes the baseline characteristics of the clinical cohort, which comprised young adults with a normal body-mass index. Per-group demographics were not formally compared, so residual demographic differences between the normal-arch and flexible-flatfoot groups cannot be excluded.

### 3.4. Gait Parameters During Standing and Walking

**Figure 7:**
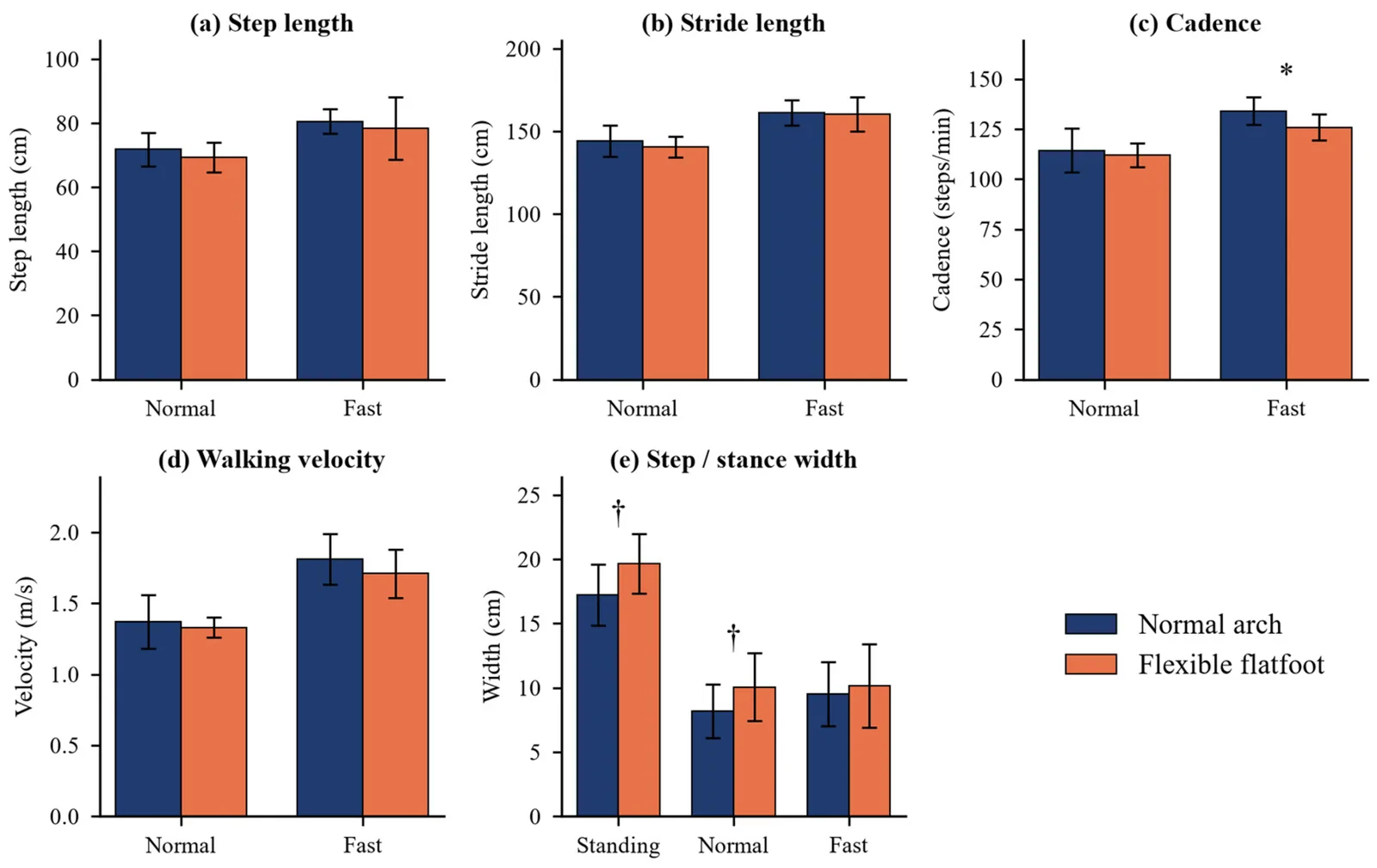
Spatiotemporal gait parameters by group and walking condition (mean ± SD; *n* = 15 per group): (**a**) Step length; (**b**) stride length; (**c**) cadence; (**d**) walking velocity; and (**e**) step width during standing, comfortable, and fast walking. * *p* < 0.05 after Holm–Bonferroni correction; † *p* < 0.05 in the uncorrected comparison only.

Clinical testing compared spatiotemporal gait parameters between the normal-arch and flexible-flatfoot groups while wearing the smart insoles. The results for standing, comfortable walking, and fast walking are presented in Table 6, with the corresponding effect sizes and statistical comparisons shown in Table 7. During static standing, the flexible-flatfoot group showed a significantly greater stance width than the normal-arch group (19.67 ± 2.32 vs. 17.22 ± 2.38 cm; +14%; Cohen’s d = 1.04; *p* = 0.008). During comfortable walking, no significant between-group differences were observed in step length, stride length, or walking velocity (all *p* > 0.18; Figure 7 and Table 7). During fast walking, step length, stride length, step width, and walking velocity also did not differ significantly between groups, whereas cadence was significantly lower in the flexible-flatfoot group (125.84 ± 6.61 vs. 133.99 ± 6.92 steps/min; −6%; Cohen’s d = 1.20; *p* = 0.003), representing the only between-group difference that remained significant after Holm–Bonferroni correction.

Step width was significantly greater in the flexible-flatfoot group during comfortable walking (10.06 ± 2.65 vs. 8.18 ± 2.07 cm; +23%; Cohen’s d = 0.79; *p* = 0.040); however, this difference was no longer significant after Holm–Bonferroni correction. During fast walking, step width remained greater in the flexible-flatfoot group (10.14 ± 3.24 vs. 9.52 ± 2.48 cm), but the between-group difference was not statistically significant (*p* = 0.561). Across all three testing conditions, mean step width was consistently greater in the flexible-flatfoot group (Figure 7e and Table 7).

## 4. Discussion

### 4.1. Interpretation of Key Findings

All study objectives were achieved. A functional smart insole was fabricated from natural-rubber composites, and its IoT monitoring system demonstrated acceptable measurement accuracy, with a maximum body-weight error of 4.18%. The materials strategy—a lightweight sponge–rubber upper combined with a durable carbon-black outsole—produced a device both comfortable and robust for daily use, demonstrating the feasibility of sustainable commodity-based biomaterials for wearable healthcare applications.

For gait, no significant between-group differences were observed in step length, stride length, or walking velocity during comfortable walking while participants wore the smart insole (all *p* > 0.18; Table 7). In contrast, cadence was significantly lower in the flexible-flatfoot group during fast walking (*p* = 0.003), representing the only between-group difference that remained significant after Holm–Bonferroni correction. These findings indicate that forward-progression parameters were comparable between groups during comfortable walking, with only cadence differing under the greater demands of fast walking. However, because both groups were evaluated only while wearing the smart insole and no barefoot or conventional-insole condition was included, the present study cannot determine whether these findings reflect the effect of the insole or the underlying gait characteristics of this young cohort with mild flexible flatfoot. Therefore, the observed similarities should not be interpreted as evidence that the smart insole normalized gait.

A consistent finding across the three testing conditions was the greater stance or step width observed in the flexible-flatfoot group. Step width was significantly greater during standing (*p* = 0.008) and comfortable walking (*p* = 0.040) in the uncorrected comparisons; after Holm–Bonferroni correction, neither remained significant—only the reduction in fast-walking cadence did—although both showed moderate-to-large effect sizes (Cohen’s d = 1.04 and 0.79, respectively). A wider base of support is a recognized compensatory strategy to enhance stability under biomechanical deficits. In flexible flatfoot, arch collapse and excessive pronation may reduce mechanical stability, prompting a wider stance or gait to maintain balance [23]. The large effect sizes for stance width during standing (d = 1.04) and step width during comfortable walking (d = 0.79), compared with the absence of significant differences in forward-progression parameters, suggest that mediolateral gait adaptations may persist despite comparable walking performance. Although this pattern may reflect a dissociation between mechanical alignment and long-established neuromuscular adaptations, this interpretation should be considered cautiously because neuromuscular function was not directly assessed and gait was not evaluated under barefoot or conventional-insole conditions.

Passive orthotic support may therefore be only one component of rehabilitation. Although supportive insoles may improve foot alignment and redistribute plantar loading, they may not immediately change motor-control strategies learned over years of altered foot mechanics. Consequently, combining orthotic support with active gait-retraining or neuromuscular rehabilitation may be a more comprehensive approach for flexible flatfoot. This interpretation is also consistent with previous reports showing that increasing step width can reduce kinematic deviations, such as excessive rearfoot eversion [23]. In contrast, the present findings suggest that the wider step width observed in individuals with flexible flatfoot may represent an intrinsic compensatory strategy that persists despite the use of a supportive insole; however, this hypothesis requires confirmation in future controlled studies.

### 4.2. Comparison with the State of the Art

The system occupies a distinctive niche. Many research and commercial insoles use denser sensor arrays and heavier computation—16 or more pressure sensors per insole for detailed plantar-pressure maps [11,24]. The present work shows instead that four strategically placed load cells achieve high accuracy (4.18% maximum error, RMSE 1.94 kg) for total and per-foot body-weight loading—comparable to or better than wearable systems reporting 1% to >15% error depending on the parameter [25,26]. Because 24-bit acquisition renders quantization negligible (Equation (5)), accuracy is bounded by mechanical coupling rather than electronics, so further gains lie in sensor–sole integration rather than added channels. The key differentiators are material and design philosophy: sustainable natural rubber departs from the synthetic polymers of most wearables and aligns with interest in eco-friendly biocomposites [17], while low-cost hardware (ESP32, minimal sensors) and a free cloud platform (ThingSpeak) make the system highly accessible. The contribution is not to outperform high-end systems on every metric, but to show that a simple, affordable, sustainably sourced system can deliver clinically relevant data for a specific application. Research- and clinical-grade plantar-pressure platforms (e.g., Tekscan F-Scan, Novel Pedar, and Moticon insoles) provide dense pressure mapping and are well validated, but they rely on synthetic materials and typically cost from several hundred to several thousand US dollars, largely confining them to specialist laboratories [11]. Consumer footwear sensors (e.g., Nurvv and Plantiga) are more affordable yet remain proprietary and are not designed around sustainable materials. The present system instead trades dense pressure mapping for total and per-foot body-load monitoring using a natural-rubber body and open, low-cost hardware, offering lower cost, greater material sustainability, and readier accessibility for remote or resource-limited settings, while remaining clinically useful for weight-bearing and symmetry monitoring.

### 4.3. Limitations

Several limitations apply. First, the dry-footprint method yields only basic spatiotemporal parameters and no kinetics (joint moments and ground reaction forces) or 3D kinematics. Second, the free ThingSpeak tier’s 15 s upload interval captures weight-bearing trends but not intra-step dynamics. Third, the 30-participant cohort is small, constraining inference. Fourth, only immediate effects were assessed, not longer-term adaptation. Fifth, and most important for the gait comparison, both groups were tested only in the supportive insole; without a barefoot or neutral-insole condition, this study is cross-sectional and cannot establish the insole’s causal effect on gait, and non-significant differences should not be read as equivalence. Sixth, all spatiotemporal parameters came from footprints, not the insole sensors—validated only as a static per-foot load monitor—so the sensing system did not itself generate the gait findings. The load-sensing chain was, moreover, characterized only under quasi-static standing loads; its accuracy for dynamic walking loads, and its drift, hysteresis, repeatability, and durability over repeated footwear-use cycles, were not evaluated and will require dedicated bench and force-plate testing. The device also reports total per-foot load rather than regional plantar-pressure distribution or discrete gait events. Seventh, the footwear adds ~400 g per foot of distal mass, a potential confounder of gait energetics. Eighth, the cohort was young (~20 years) and narrow, and the flatfoot classification criterion should be specified, limiting generalization to pediatric and older symptomatic flatfoot. Finally, with 11 comparisons and 15 per group, this is best treated as an effect-size/pilot study; confirmatory work should pre-register hypotheses, correct for multiple comparisons (e.g., Holm–Bonferroni), and use equivalence testing where ‘no difference’ is claimed.

### 4.4. Future Directions

These limitations point to clear next steps. Integrating IMUs with the pressure sensors would enable sensor fusion and full 3D lower-limb kinematics [9,12]. On-board microSD logging would allow for high-frequency capture, and the resulting datasets could train machine-learning models—e.g., long short-term memory (LSTM) networks—to detect gait events (heel strike and toe-off) and classify gait patterns [24,27]. Longitudinal studies over weeks to months would show whether the compensatory wide step width adapts, informing neuromuscular plasticity and rehabilitation. Finally, the platform could, after dedicated validation in each group, be explored in other populations that may benefit from remote gait and load monitoring—diabetic neuropathy (ulcer prevention), post-stroke rehabilitation, and Parkinson’sdisease [27]. A further priority is miniaturization: replacing the discrete ESP32 board and separate enclosure with a system-on-chip or flexible printed circuit embedded within the sole, together with a smaller rechargeable cell, would reduce the added mass and bulk, improving comfort and enabling unobtrusive long-term and remote monitoring. Bench characterization of the sensing chain is the most immediate priority: cyclic loading over at least 10^4^ cycles at physiological load levels to quantify any change in sensitivity or zero offset, loading–unloading sweeps to quantify hysteresis, sustained loading to quantify drift, and repeated don–doff and across-day measurements to quantify repeatability. Dynamic performance should then be established by comparing the insole output with a calibrated force plate or instrumented treadmill during walking, which would also allow gait-event detection to be validated against a reference system.

## 5. Conclusions

This work presented the design, fabrication, and multi-faceted validation of a smart insole for remote body-load monitoring. Combining natural-rubber composites with a low-cost, accessible IoT architecture, the device measures total and per-foot body-weight loading with a mean absolute error of 2.94% (RMSE 1.94 kg) and a maximum error of 4.18%, the residual being an almost purely systematic, single-parameter-correctable bias. The approach offers a pathway for turning a primary national commodity into high-value medical technology, addressing economic and healthcare goals together. Clinically, no significant between-group differences were observed in forward-progression parameters during comfortable walking while participants wore the smart insole, whereas greater stance and step width remained evident in the flexible-flatfoot group. Because gait was evaluated only while wearing the smart insole and no barefoot or conventional-insole comparison was included, these findings should not be interpreted as evidence of the therapeutic effect of the insole. Future controlled studies are warranted to determine whether combining orthotic support with active gait retraining can further optimize gait performance. The platform also supports future enhancement through sensor fusion and machine learning, and extension to broader populations needing continuous remote gait monitoring.

## Figures and Tables

**Table 1:** Final natural-rubber composite formulations (parts per hundred rubber, phr).

Ingredient	Upper Sponge Sole (Si-20-OBSH-4)	Lower Solid Sole (C-30)
Natural rubber (STR 5L)	100	100
Silica (Tokusil 255G)	20	–
Carbon black (HAF N-330)	–	30
Silane coupling agent (Si69)	1.77	–
Zinc oxide (ZnO)	5	5
Stearic acid	1	1.5
BHT (antioxidant)	1	–
TMQ (antioxidant)	–	1
DPG (accelerator)	1.1	1
CBS (accelerator)	1.2	0.6
OBSH (blowing agent)	4	–
Kicker (activator)	1.2	–
Titanium dioxide (TiO_2_)	1	–
Pigment	1	–
Sulfur	2.5	2.5

**Table 2:** Principal hardware and cloud components of the smart insole system.

Subsystem	Component	Key Specification/Role
Force sensing (final)	4 × load cell per insole	Strain-gauge transducers at the calcaneus, medial and lateral metatarsal heads, and hallux
Load-cell amplifier	HX711 24-bit ADC (one per foot)	Combines the four load cells (Wheatstone bridge) and reports the per-foot load to the ESP32 over a two-wire (DAT/CLK) interface
Force sensing (early prototype)	FlexiForce piezoresistive sensor (Tekscan) + resistance-to-voltage module	Converts sensor resistance to a voltage read by the ESP32’s on-chip ADC
Processing and radio	ESP32 (ESP-32 Mini32 V2.0.13)	Tensilica LX6 dual-core 240 MHz, 520 KB SRAM; Wi-Fi 802.11 b/g/n and Bluetooth 4.2 BLE; 12-channel ADC; programmed in Arduino IDE
User feedback	0.96-inch OLED display (I^2^C)	Wrist-worn real-time display of per-foot load
Interconnect and enclosure	I^2^C serial bus; 4.5 × 4.5 × 2 cm housing	Minimal wiring between modules; compact wearable package
Power	Li-polymer cell (502530), 3.7 V/370 mAh	Rechargeable; compact form factor for wearable use
Cloud platform	ThingSpeak (Wi-Fi, 15 s upload)	Field 1 (left) and Field 2 (right); live plots, storage, CSV export

**Table 3:** Measured properties of the two natural-rubber compounds at the selected filler loadings (mean values before heat aging).

Property	Standard	Si-20 (Silica, 20 phr)	C-30 (Carbon Black, 30 phr)
Hardness (Shore A)	ASTM D2240	50	55
300% modulus (MPa)	ASTM D412	5.0	9.6
Tensile strength (MPa)	ASTM D412	34	31
Elongation at break (%)	ASTM D412	750	555
Tear strength (N/mm)	ASTM D624	63	104

**Table 4:** Summary of smart insole system performance.

Performance Indicator	Value
Validation participants (n)	25
Correlation with reference scale (Pearson r)	0.99
Mean absolute error	2.94%
Maximum absolute error	4.18%
Load cells per insole	4
Cloud upload interval	15 s
Approximate device mass	≈400 g per shoe
Root-mean-square error	1.94 kg
Calibration line slope/R^2^	0.966/0.980
Systematic gain, k (proportional bias)	0.97
Mean error after scalar recalibration	0.47%
Effective load resolution (24-bit ADC)	<0.3 g
Mean absolute weight-bearing asymmetry	8.8%

**Table 5:** Baseline characteristics of the clinical cohort(*n* = 30; normal arch, *n* = 15; flexible flatfoot, *n* = 15). Values are pooled across both groups.

Characteristic	All Participants (Mean ± SD)
Age (years)	20.29 ± 1.21
Body mass (kg)	59.70 ± 9.70
Height (cm)	161.60 ± 5.14
BMI (kg/m^2^)	22.81 ± 3.11

**Table 6:** Spatiotemporal gait parameters by group and walking condition (mean ± SD; *n* = 15 per group) for the normal-arch and flexible-flatfoot groups during standing, comfortable (normal) walking, and fast walking. Dashes (–) denote parameters not applicable to the standing condition. For the standing condition, step width denotes the mediolateral stance width (distance between the feet).

Gait Parameter	Group	Standing	Normal Walk	Fast Walk
Step length (cm)	Normal	–	71.78 ± 5.31	80.54 ± 3.85
	Flatfoot	–	69.32 ± 4.59	78.36 ± 9.76
Stride length (cm)	Normal	–	144.27 ± 9.42	161.34 ± 7.54
	Flatfoot	–	140.66 ± 6.29	160.21 ± 10.42
Step width (cm)	Normal	17.22 ± 2.38	8.18 ± 2.07	9.52 ± 2.48
	Flatfoot	19.67 ± 2.32	10.06 ± 2.65	10.14 ± 3.24
Cadence (steps/min)	Normal	–	114.36 ± 11.07	133.99 ± 6.92
	Flatfoot	–	111.89 ± 6.03	125.84 ± 6.61
Velocity (m/s)	Normal	–	1.37 ± 0.19	1.81 ± 0.18
	Flatfoot	–	1.33 ± 0.07	1.71 ± 0.17

**Table 7:** Between-group statistical comparison of spatiotemporal gait parameters (flexible flatfoot vs. normal arch; n = 15 per group). Positive differences denote larger values in the flatfoot group; d is Cohen’s effect size, and *p* is from Welch’s *t*-test. Bold *p*-values are significant (p< 0.05).

Parameter	Condition	Difference *	Cohen’s d	Welch t	p
Step length (cm)	Normal	−3.4%	0.50	1.36	0.186
	Fast	−2.7%	0.29	0.80	0.431
Stride length (cm)	Normal	−2.5%	0.45	1.23	0.229
	Fast	−0.7%	0.12	0.34	0.736
Velocity (m/s)	Normal	−2.9%	0.28	0.77	0.454
	Fast	−5.5%	0.57	1.56	0.129
Cadence (steps/min)	Normal	−2.2%	0.28	0.76	0.456
	Fast	−6.1%	1.20	3.30	**0.003**
Step width (cm)	Standing	+14.2%	1.04	2.85	**0.008**
	Normal	+23.0%	0.79	2.17	**0.040**
	Fast	+6.5%	0.21	0.59	0.561

* Difference computed as (flatfoot–normal)/normal × 100% from the group means in Table 6.

## Data Availability

The data presented in this study are available on request from the corresponding author. The data are not publicly available because they contain information that could compromise the privacy of the research participants.
